# Microstructural Characteristics and Subsequent Soften Mechanical Response in Transverse Direction of Wrought AZ31 with Elevated Compression Temperature

**DOI:** 10.3390/ma14144055

**Published:** 2021-07-20

**Authors:** Mengmeng Yang, Feng Zhang, Wei Yu, Yikui Bai, Zheng Liu

**Affiliations:** 1 School of Water Conservancy, Shenyang Agricultural University, Shenyang 110866, China; 20096011@mmm.muroran-it.ac.jp (M.Y.); Fzhang2019@syau.edu.cn (F.Z.); baiyikui@syau.edu.cn (Y.B.); 2 Muroran Institute of Technology, Division of Engineering, Muroran 050-8585, Japan; 3 School of Material Science and Engineering, Shenyang University of Technology, Shenyang 110870, China; cl0804zf@126.com

**Keywords:** AZ31-TD, dynamic compression, deformation mechanism, CRSS, SF

## Abstract

In order to investigate the effect of temperature on the microstructure evolution and mechanical response in the transverse direction of a wrought AZ31 (AZ31-TD) alloy under a high strain rate, the dynamic compression was conducted using Split Hopkinson Pressure Bar (SHPB) apparatus and a resistance-heated furnace under 1000 s^−1^ at 20–250 °C. By combining optical and EBSD observations, the microstructure’s evolution was specifically analyzed. With the help of theoretically calculated Schmid Factors (SF) and Critical Resolved Shear Stress (CRSS), the activation and development deformation mechanisms are systematically discussed in the current study. The results demonstrated that the stress–strain curves are converted from a sigmoidal curve to a concave-down curve, which is caused by the preferentially and main deformation mechanism $\left\{10\bar{1}2\right\}$ tension twinning gradually converting to simultaneously exist with the deformation mechanism of a non-basal slip at an elevated temperature, then completing with each other. Finally, the dynamic recrystallization (DRX) and non-basal slip are largely activated and enhanced by temperature elevated to weaken the $\left\{10\bar{1}2\right\}$ tension twinning.

## 1. Introduction

Mg is the eighth most common element in the crust of the earth, which is also extractable from seawater [1]. With the requirement of a lightweight, energy-efficient and environmentally benign system, the magnesium alloy was widely used in an environment where the science of material design processing could now address its complexities and offer up new possibilities [2,3]. Due to the hexagonal close-packed (HCP) crystalline structure, only limited independent slip systems of magnesium and its alloy could be initially activated at room temperature [4]. Hence, the mechanical properties of magnesium and its alloy were low formability, limited ductility and premature failure no mater quasi-static or dynamic deformation [5,6,7]. However, compared with room temperature conditions, the deformation mechanisms of magnesium and its alloys were significantly different and complex under high temperatures [8,9,10,11,12,13,14,15,16,17,18,19]. According to the extreme requirement of the magnesium alloy structure application, the knowledge of their mechanical response and microstructure evolution under a high strain rate and elevated temperature were significantly important [20,21,22].

In recent years, considerable effort has been made to research the deformation mechanisms of magnesium and its alloy under elevated temperature conditions, especially a wrought magnesium alloy. Generally, fracture strain (strain to failure) increases with increasing experimental temperature, whereas fracture strain decreases with the increasing experimental strain rate [23]. Hence, the temperature increase was known to effectively soften the mechanical properties of the magnesium alloy. The strain rate increase was known to enhance the flow stress and weaken the fracture strain. However, in the research of a solution-treated cast AM80 magnesium alloy with the strain rate ranging from 1100 to 5000 s^−1^ at 298, 423 and 523 K, the effect of the strain rate on the fracture strain varied from enhanced to weakened as the strain rate increased to its critical value at all the temperature conditions [16]. It was found that the negative strain rate sensitivity was mainly caused by adiabatic shear bands (ASBs) and dynamic recrystallization (DRX). The ASBs were generated at 298 K under a strain rate of 5000 s^−1^. With elevated temperatures of 423 and 523 K, recrystallized microstructure forms were gradually generated on the basic of twins and dislocations, which were nucleated at the twins’ boundaries and extended between the twins’ boundaries and grains’ boundaries. As a typical wrought magnesium alloy, the mechanical properties of an AZ31 rolled sheet were strongly anisotropic, which was caused by typical {0002} texture [5,24,25]. During the dynamic compressive deformation in a strain rate range from 10^−4^ to 3500 s^−1^ at room temperature, the increased fracture strain was 30% in the transverse direction (TD) and 55% in the rolling direction (RD) under 3500 s^−1^ as compared to 10^−4^ s^−1^ [20]. The twinning was the main deformation mechanism in the rolled direction, while the slipping seemed to be a significantly influenced participant in the 45° and normal direction. The flow stress behavior, strongly dependent on the strain rate, increased in the normal direction (ND). However, the strong strain rate dependence for compressive yield stress was not obviously exhibited in TD and RD [26]. For the AZ31 rolled sheet during the dynamic compression and tension behavior at the strain rates of ~10^3^ s^−1^, the activation of $\left\{10\bar{1}2\right\}$ tension twinning was extremely enhanced by the increased strain rate, while there was no significant strain rate dependence in contraction and secondary twinning [27]. In fact, the deformation behavior of the AZ31 rolled sheet was dependent on not only the strain rate but also the temperature. The typical result was shown that the anisotropy of the AZ31 rolled sheet, including the yield stress, peak stress and microstructure, was gradually weakened with the increased temperature [28]. Until the temperature was close to 350 °C, the anisotropy of the AZ31 rolled sheet almost disappeared. To sum up, most of the investigations on the anisotropy of an AZ31 rolled sheet were only focused on the activation and evolution of $\left\{10\bar{1}2\right\}$ tension twinning, non-basal slip and dynamic recrystallization mechanisms by changing temperatures. The detailed discussion about the relationship between twins and dynamic recrystallization mechanisms in a wrought AZ31 sheet were very limited, even unreported.

In our current study, the dynamic compression test of a wrought AZ31-TD sheet was conducted by split Hopkinson pressure bar (SHPB) and resistance-heated furnace at 1000 s^−1^ strain rate under 20–350 °C temperature conditions. With the temperature elevated, the evolution of the microstructure mechanism was analyzed, especially the relationship between $\left\{10\bar{1}2\right\}$ tension twinning and dynamic recrystallization (DRX). Finally, the effect of temperature on the microstructural characteristics and subsequent soften mechanical responses of the wrought AZ31-TD sheet were systematically discussed by electron backscattered diffraction (EBSD), Schmid Factors (SF) and Critical Resolved Shear Stress (CRSS).

## 2. Experimental

### 2.1. Experimental Material

The wrought AZ31-TD sheet was offered by a Canada–China–USA collaborative research and development project, MAGNESIUM FRONT END RESEARCH AND DEVELOPMENT (MFERD), which was produced by Timminco Metals in Denver, CO, USA. The chemical composition of the AZ31 magnesium alloy is listed in Table 1.

The wrought AZ31 sheet (with an average grain size ~25 μm) was proceeded by multi pass under 450 °C with a thickness of 8 mm. In order to avoid the negative effect on the microstructure mechanism analysis by the initial microstructure, the heat treatment process of detwinning was 300 °C × 2 h.

### 2.2. Experimental Method

**Figure 1:**
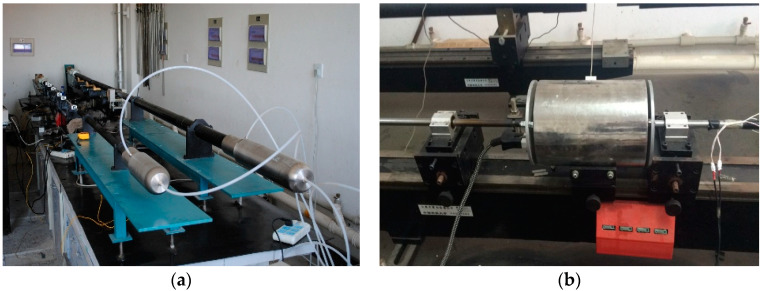
SHPB apparatus with resistance-heated furnace: (**a**) SHPB apparatus, (**b**) resistance-heated furnace.

The dynamic compression tests were conducted under 1000 s^−1^ strain rate with a temperature from 20 °C to 250 °C by combining SHPB apparatus and a resistance-heated furnace, which are shown in Figure 1.

The strain rate was controlled by impactive pressure and stroking on striker in pumping chamber. Additionally, the temperature was measured using a thermocouple in the resistance-heated furnace. The dynamic compression test experiment was conducted after 10 min of soaking time. The size of cylindrical sample was Φ6 × 6 mm, which was cut along the TD of the wrought AZ31 sheet by using an electrical discharged wire-cutter. At least 3 samples were repeated for every experimental condition to avoid error influence. After comparing the test result of every experiment, the stress–strain curves of the wrought AZ31-TD sheets were hardly influenced by the error. The cross sections of the cylindrical samples were polished using standard metallographic techniques, and then etched for 5~10 s in acetic picral (100 mL ethanol, 10 mL H_2_O, 6 g picric acid, 5 mL acetic acid). The optical microscope was conducted using a ZEISS metallographic microscope (Axio vert A1m). The microstructure and texture analysis were performed by using a field emission MERLIN Compact and scanning electron microscope (SEM), equipped with an EBSD detector system. In order to obtain a clear EBSD observation, it was necessary to conduct electrolysis treatment on the post impact samples. The etch solution was comprised of 4.2 g picric acid, 10 mL acetic acid, 10 mL H_2_O and 70 mL ethanol.

## 3. Result

### 3.1. Dynamic Mechanics Response

**Figure 2:**
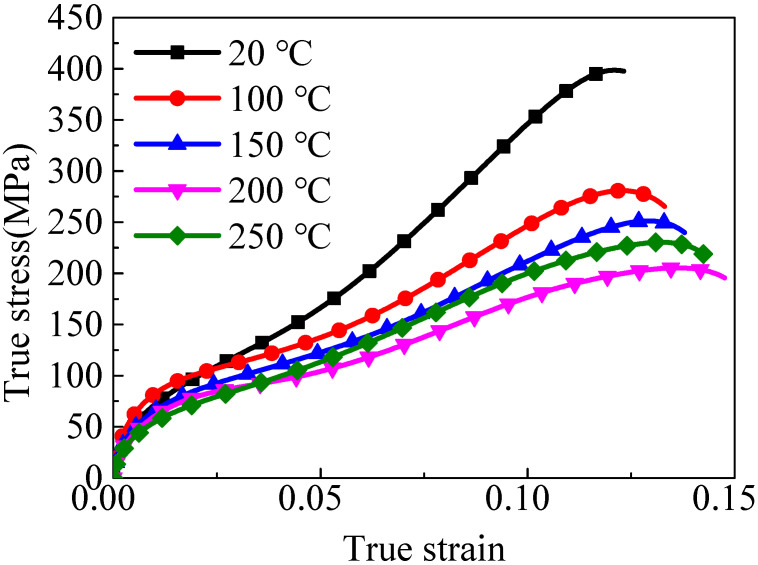
Stress–strain curves at different temperatures under 1000 s^−1^.

The stress–strain curves of the wrought AZ31-TD alloy with an elevated temperature under 1000 s^−1^ are presented in Figure 2.

The dynamic mechanics response of the wrought AZ31-TD alloy at room temperature is obviously highly anisotropic, which is shown by the sigmoidal curve. As the direction of impact loading is perpendicular to the normal direction of {0002} texture plan, it is easier for a large number of $\left\{10\bar{1}2\right\}$ tension twinning to be activated, leading to the typical characteristic curve presence [29]. With the temperature increased, the critical strain, corresponding to the peak stress, gradually increases from 0.12 to 0.14. The peak stress decreases from 398.61 to 204.16 MPa with temperature increased. Finally, the evolution trend of stress–strain curves are from a sigmoidal curve to a concave-down curve. It is illustrated that the thermal softening has large effect on the wrought AZ31-TD dynamic deformation behavior. This phenomenon is caused by the reduced critical resolved shear stress (CRSS) of the non-basal slip and more activation of the non-basal glide systems, which is similarly reported by Liu et al. [21].

### 3.2. Microstructure Characteristic

**Figure 3:**
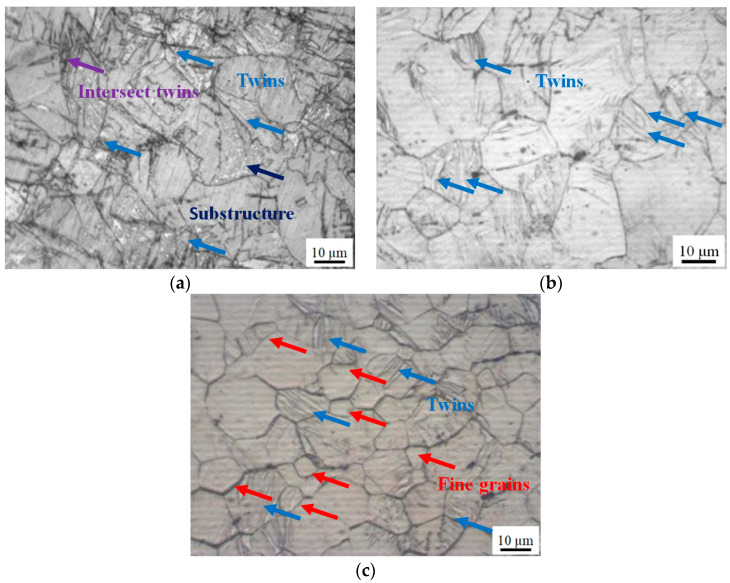
Microstructure in elevated temperature under 1000 s^−1^: (**a**) 20 °C, (**b**) 150 °C, (**c**) 250 °C.

The microstructures of the samples with an elevated temperature under 1000 s^−1^ are shown in Figure 3.

In all the temperature conditions, a large number of twins are present at 1000 s^−1^, which partly consist of double twins and intersect twins. Meanwhile, the substructures are observed at 150 °C, which are randomly dispersed in the large grains. By comparing Figure 3a,b, it can be obviously seen that the twins gradually thickened and more dense with the increased temperature. Additionally, the grains are gradually separated by the twins. This phenomenon is usually caused by twinning dislocation spread on adjacent planes [20]. In addition, the c-axis, the normal direction of {0002} plan, is perpendicular to the loading direction, which is also a benefit for $\left\{10\bar{1}2\right\}$ tension twinning activation. Additionally, the twins’ boundaries generated in the same grains are usually parallel to each other [29]. In Figure 3c, a large number of refinement grains are present, with grains of 3–4 μm radius size. On one hand, the twins completely disappeared as a result of detwinning occurring in some grains. On the other hand, the density and thickness of the twins are further increased. As fine grains induce slip deformation more easily, twinning is hardly activated. However, when the refinement grains are grown to be coarse grains by the elevated temperature, the formation of twinning is more easily activated [28]. Hence, the refinement grains may have been caused by dynamic recrystallized (DRX) as the temperature increased. Additionally, the twins in large grains may be present after the refinement grains.

## 4. Discussion

### 4.1. Effect of Temperature on Microstructure Evolution

**Figure 4:**
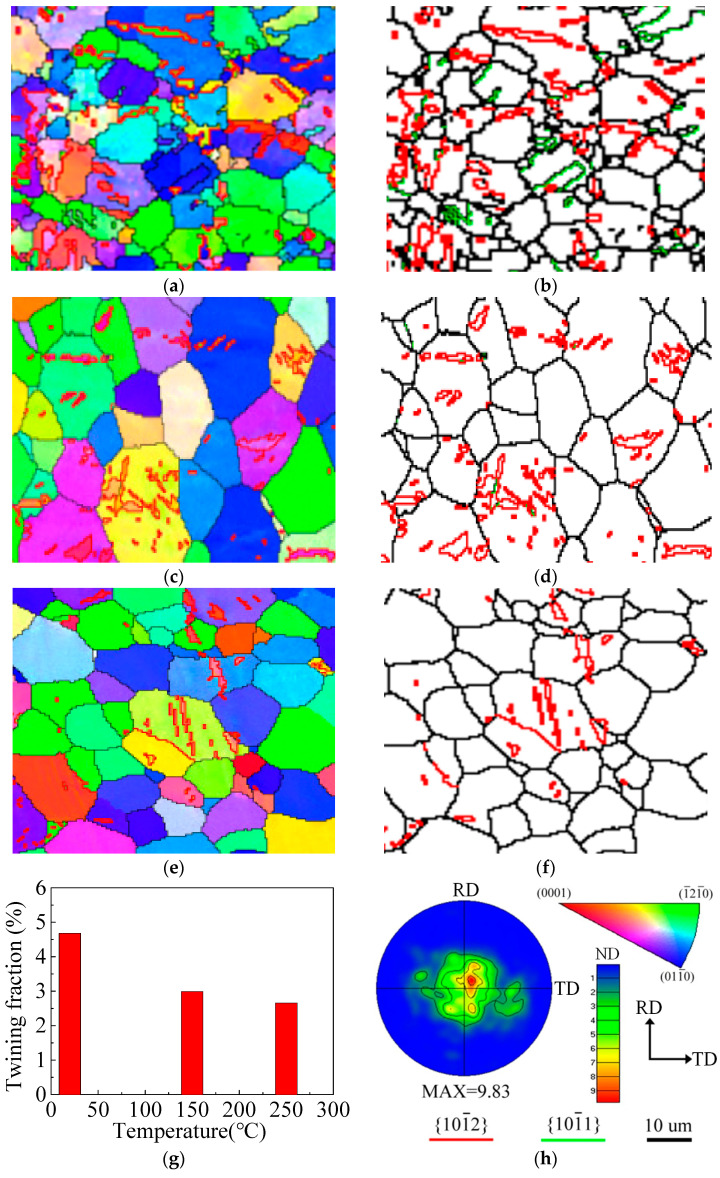
IPF maps, PF maps, BM maps and TVF of impacted wrought AZ31-TD alloy: (**a**) IPF at 20 °C, (**b**) BM at 20 °C, (**c**) IPF at 150 °C, (**d**) BM at 150 °C, (**e**) IPF at 250 °C, (**f**) BM at 250 °C, (**g**) twinning fraction under different temperature condition, (**h**) PF at 20 °C.

The inverse pole figure (IPF) maps, pole figure (PF), boundaries misorientation (BM) and twins’ volume fraction (TVF) of the wrought AZ31-TD alloy after impact are shown in Figure 4, which was obtained from the EBSD data collected. In Figure 4, the black line is the grain boundary. The red line and green lines are the $\left\{10\bar{1}2\right\}$ tension twin boundary and $\left\{10\bar{1}1\right\}$ contraction twin boundary, respectively, which is illustrated and corresponds to the red line and green line in Figure 4h.

**Figure 5:**
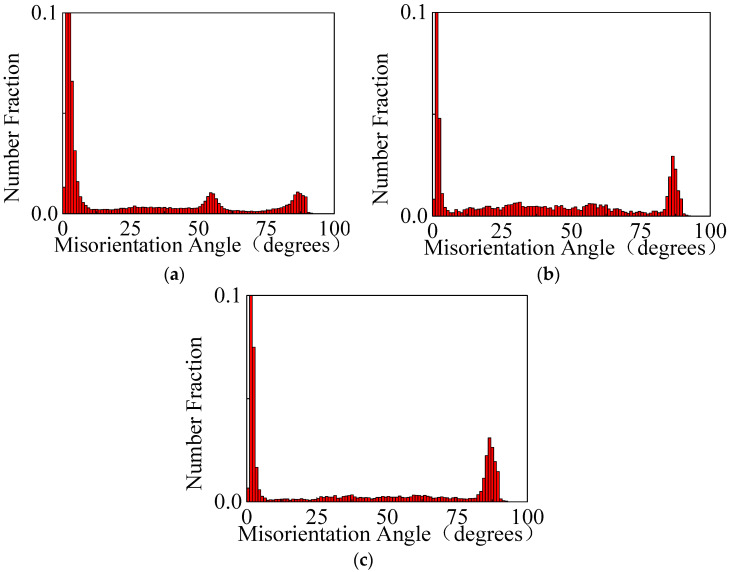
Misorientation angle distribution of impacted wrought AZ31-TD alloy: (**a**) 20 °C, (**b**) 150 °C, (**c**) 250 °C.

By combining Figure 4a,b,g, the TVF of the wrought AZ31-TD alloy at a temperature of 20 °C is 4.68%, which consists of $\left\{10\bar{1}1\right\}$ contraction twins and $\left\{10\bar{1}2\right\}$ tension twins. The three type of twins, such as $\left\{10\bar{1}2\right\}$ tension twins, $\left\{10\bar{1}1\right\}$ contraction twins and $\left\{10\bar{1}1\right\}-\left\{10\bar{1}2\right\}\;$double twins, are determined by the particular misorientation angles and rotation axis of twins [30]. In detail, the characteristics of a double twins-matrix, tension twins-matrix and contraction twins-matrix are the misorientation of 38° about $\langle \bar{1}2\bar{1}0\rangle$, 56° about $\langle \bar{1}2\bar{1}0\rangle$ and 80° about $\langle \bar{1}2\bar{1}0\rangle ,\;\mathrm{respectively}$ [21]. In order to further identify the type of twins, the misorientation angle distribution of the impacted wrought AZ31-TD alloy at different temperatures is shown in Figure 5.

It can been seen that the misorientation angle distributions are, respectively, concentrated in 38° about $\langle \bar{1}2\bar{1}0\rangle ,$ 56° about $\langle \bar{1}2\bar{1}0\rangle$ and 80° about $\langle \bar{1}2\bar{1}0\rangle$. In addition, a little of the misorientation angle distribution is located in 38° about $\langle \bar{1}2\bar{1}0\rangle$; $\left\{10\bar{1}1\right\}-\left\{10\bar{1}2\right\}$ double twins also exist in the matrix of the wrought AZ31-TD alloy. When the experimental temperature is increased to 150 °C, the most frequently observed boundaries are those with a misorientation of 80° about $\langle \bar{1}2\bar{1}0\rangle$. Additionally, almost none of 56° about $\langle \bar{1}2\bar{1}0\rangle$ misorientation boundaries are found in Figure 4c,d, which also corresponds to the misorientation angle distribution in Figure 5b. Meanwhile, the TVF of the wrought AZ31-TD alloy is decreased to 2.98%, as shown in Figure 4g. This phenomenon may be caused by detwinning occurring at a high temperature, which has been addressed in previous research [19]. It is also illustrated that the predominant mechanism of non-uniform deformation with twinning is gradually weakened. As the stress is concentrated at a dislocation pile-up at the grain and twin boundaries, the non-basal slip, such as the pyramidal slip <a> and the pyramidal slip <c + a>, can be activated, including the boundary regions and the mantle region. Hence, the size of grain is relatively smaller. In this way, the stress concentration is relaxed, leading to the suppression of the $\left\{10\bar{1}1\right\}$ contraction twins and $\left\{10\bar{1}2\right\}$ tension twins [31]. Furthermore, when the experimental temperature is increased to 250 °C, almost all of the $\left\{10\bar{1}1\right\}-\left\{10\bar{1}2\right\}$ double twins and $\left\{10\bar{1}1\right\}$ contraction twins disappear, as shown in Figure 4e–g. The TVF of the wrought AZ31-TD alloy is directly decreased to 2.66%. According to Figure 4f and Figure 5c, only a few of the $\left\{10\bar{1}2\right\}$ tension twins are located in the matrix of the wrought AZ31-TD alloy. Additionally, grains refinement is gradually present, which is caused by dynamic recrystallization. On one hand, as the large number of dislocations are easily slipped and piled up to the grain boundaries with an increased temperature, and the original grain boundaries act as the block, the DRX mechanism is initially activated at the grain boundaries due to the density of the dislocation rising to a threshold. Particularly, the stacking fault energy of the wrought AZ31-TD alloy is relatively lower. Then, the continuous DRX mechanism is enhanced by elevating the temperature beyond 250 °C [32]. On the other hand, the DRX can be also induced by strain under low temperature (110 °C) and medium temperature (210 °C) in two types of magnesium alloy during the compression process [33]. (i) When the numerous twins, dislocation and twin-twin intersection exist simultaneously at a low temperature, the DRX grains initially nucleate in the twins and the twin–twin intersection, then gradually expand outward from the inside of the original grains with an increased temperature. (ii) Compared with the DRX mechanism of a low temperature, the DRX grains initially nucleate around the original grain boundaries, which are induced by a larger strain. With the strain elevated, the DRX grains are expanded and even continuously distributed to the interior of the original grains.

### 4.2. Effect of Temperature on Dynamic Compressive Behavior

**Figure 6:**
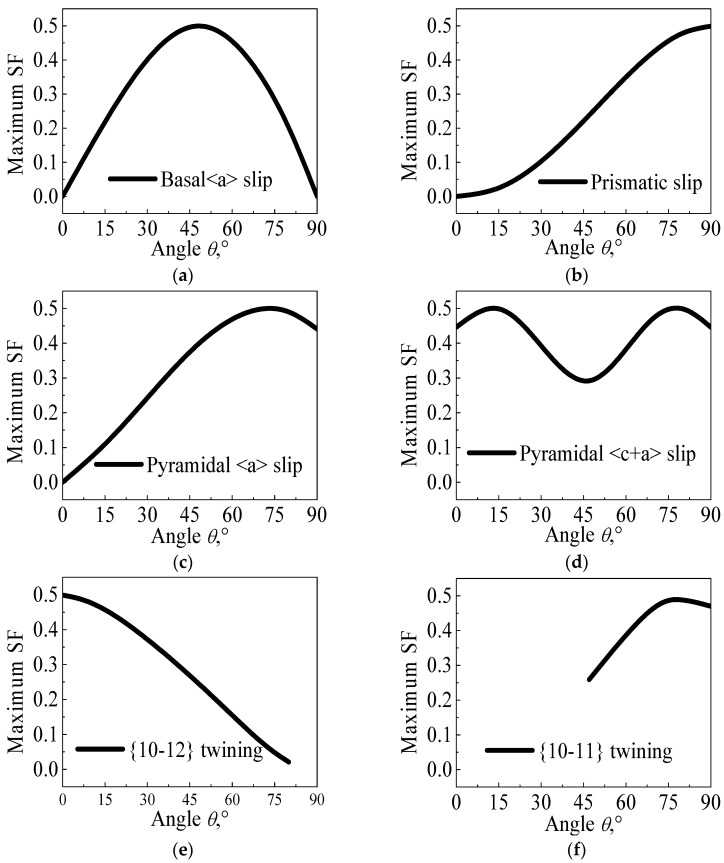
θ-maximum SF curves under different microstructure deformation mechanisms, (**a**) basal <a> slip, (**b**) prismatic slip, (**c**) pyramidal <a> slip, (**d**) pyramidal <c + a> slip, (**e**)$\;\left\{10\bar{1}2\right\}\;\mathrm{tension}$ twinning, (**f**) $\left\{10\bar{1}1\right\}$ contraction twinning.

During the dynamic compressive deformation, the mechanical response of the wrought AZ31-TD alloy is directly determined by microstructure mechanism evolution. As the AZ31 magnesium alloy is the low symmetry of hexagonal close-packed (HCP) structure, five independent slip systems exist to satisfy the requirement of the critical stress in homogeneous deformation, especially $\left\{10\bar{1}1\right\}$ contraction twinning and $\left\{10\bar{1}2\right\}$ tension twinning. Generally, $\left\{10\bar{1}2\right\}$ tension twinning is induced by a tension loading direction parallel to the c-axis or a contraction loading direction perpendicular to the c-axis. However, the type of $\left\{10\bar{1}1\right\}$ contraction twinning induced is exactly opposite to the type of $\left\{10\bar{1}2\right\}$ tension twinning [34]. Additionally, the critical stress of the microstructure mechanism activation, which is almost dependent on the critical resolved shear stress (CRSS) and the Schmid factors (SF) [28,35,36], is given as follows:

(1) $$\sigma =\frac{\tau_{CRSS}}{m}$$

where σ stands for the critical stress of microstructure mechanism activation, $\tau_{CRSS}$ stands for CRSS, m stands for SF. Additionally, the SF is established as follows:

(2) m=cosφ⋅cosλ

where φ stands for the angle between the loading direction and the twinning (or slip) plane normal and λ stands for the angle between the loading direction and the twinning (or slip) direction. For the indices direction of the two-four-dimensional Miller–Bravais system, such as {h,k,i,l}−〈u,v,t,w〉, the twinning or slip plane normal can be calculated as follows:

(3) $$\left[u,v,t,w\right]=\left[h,k,i,\frac{3l}{2}{\left(\frac{c}{a}\right)}^{2}\right]$$

where a and c are the lattice constants and, generally, the c/a axial ratio is 1.624 [37]. Finally, the cosφ and cosλ can be directly calculated using the follow equation:

(4) $$\cos\varphi (\lambda )=\frac{V_{1}\cdot V_{2}}{\left|V_{1}\right|\cdot \left|V_{2}\right|}=\frac{u_{1}u_{2}+V_{1}V_{2}+\frac{1}{2}(u_{1}V_{2}+u_{2}V_{1})+\frac{1}{3}w_{1}w_{2}{\left(\frac{c}{a}\right)}^{2}}{\sqrt{u_{1}{}^{2}+V_{1}{}^{2}+u_{1}V_{1}+\frac{V_{1}{}^{2}}{3}{\left(\frac{c}{a}\right)}^{2}}\times \sqrt{u_{2}{}^{2}+V_{2}{}^{2}+u_{2}V_{2}+\frac{V_{2}{}^{2}}{3}{\left(\frac{c}{a}\right)}^{2}}}$$

where *V*_1_ $\left[u_{1},v_{1},t_{1},w_{1}\right]$ is the twinning (slip) plane normal or twinning (slip) direction and *V*_2_ $\left[u_{2},v_{2},t_{2},w_{2}\right]$ is the loading direction. According to Equations (2)–(4), the relationship between the SF value and θ about the different microstructure deformation mechanisms, such as the basal <a> slip, prismatic slip, pyramidal <a> slip, pyramidal <c + a> slip,$\;\left\{10\bar{1}2\right\}\;\mathrm{tension}$ twinning and $\left\{10\bar{1}1\right\}$ contraction twinning, can be calculated as shown in Figure 6.

Additionally, the θ stands for the angle between the loading direction and the c-axis. The CRSS for the microstructure deformation mechanism of the wrought AZ31-TD alloy was predicted by Barnett, based on the Taylor model at 20, 150 and 250 °C, which is listed in Table 2 [38,39,40,41].

**Figure 7:**
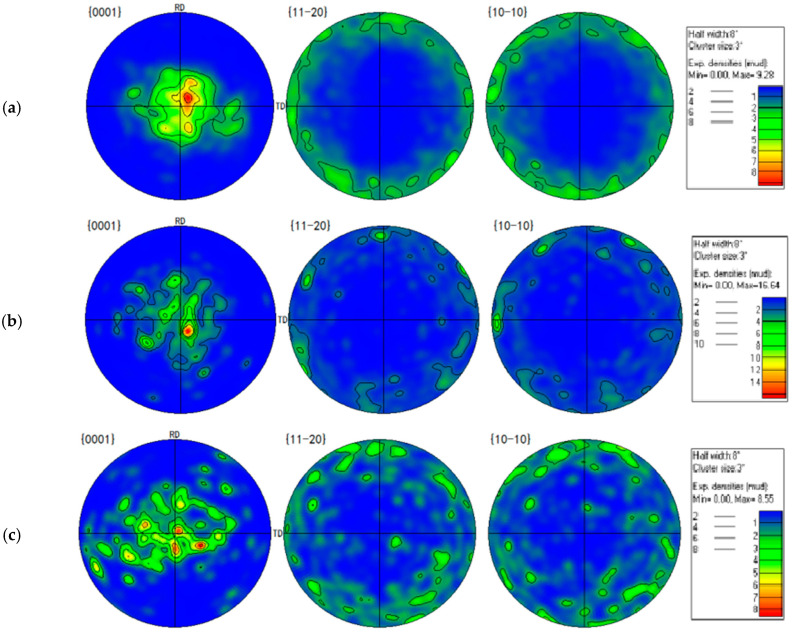
The PF maps of wrought AZ31-TD alloy at 1000 s^−1^ under different temperatures: (**a**) 20 °C, (**b**) 150 °C, (**c**) 250 °C.

According to Table 2, the CRSS of the basal slip, prismatic slip, pyramidal <c + a> slip and $\left\{10\bar{1}1\right\}$ contraction twinning presents its tendency to decrease with the temperature increasing from 20 to 250 °C. However, the evolution tendency of CRSS for $\left\{10\bar{1}2\right\}$ tension twinning is the opposite. The PF maps of the wrought AZ31-TD alloy under different temperature conditions are shown in Figure 7.

**Figure 8:**
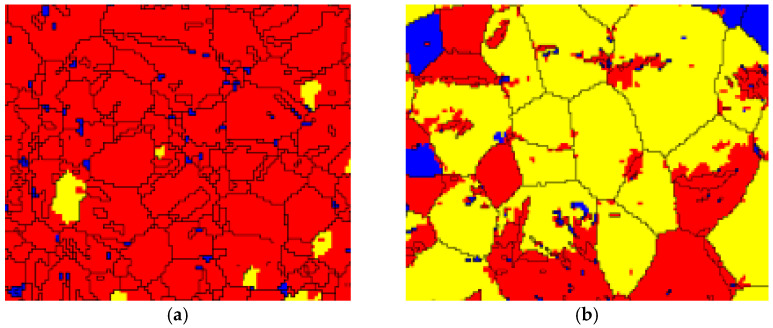

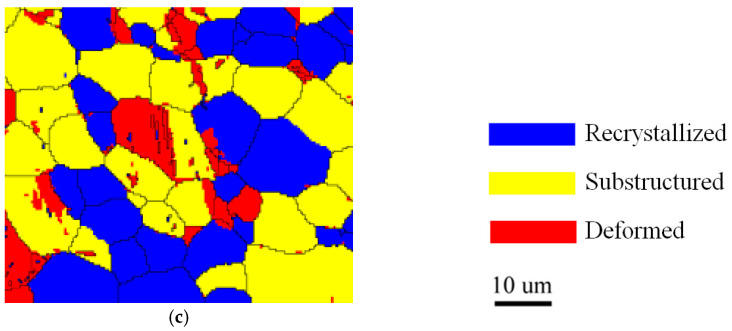
Distribution maps of the recrystallized, substructured and deformed grains: (**a**) 20 °C, (**b**) 150 °C, (**c**) 250 °C.

Hence, when the impact experiment is conducted at 20 °C, and the compressive impacting direction is perpendicular to the c-axis, the tension loading direction is particularly parallel to the c-axis. Moreover, the SF values of the prismatic slip, pyramidal <a> slip, pyramidal <c + a> slip and $\left\{10\bar{1}2\right\}$ tension twinning are approximate to 0.5, according to a combination of Figure 6 and Figure 7a. According to Equation (1) and Table 2, the σ values of the prismatic slip, pyramidal <c + a> and $\left\{10\bar{1}2\right\}$ tension twinning are almost approximate to 180, 220 and 60 MPa, respectively. Hence, the $\left\{10\bar{1}2\right\}$ tension twinning is a priority to be activated. Additionally, the stress–strain curve presents a sigmoidal curve, as shown in Figure 2. When the temperature of the experiment is 150 °C, the direction of the c-axis partly departs from the normal direction of the wrought AZ31 alloy sheet after post impact, as shown in Figure 7b. Meanwhile, the CRSS values of the prismatic slip, pyramidal <c + a> and $\left\{10\bar{1}2\right\}$ tension twinning are 60, 65 and 32 MPa, respectively. The σ of some prismatic slips and pyramidal <c + a> may be equivalent or even smaller than the σ of $\left\{10\bar{1}2\right\}$ tension twinning. Hence, the priority extent of $\left\{10\bar{1}2\right\}$ tension twinning is gradually weakened. It is illustrated that not only does the $\left\{10\bar{1}2\right\}$ tension twinning play an important role but the dislocation slip is also very important in the deformation mode during dynamic compression at 150 °C. In addition, the deformation mechanism is usually activated by twinning induced in a grain due to the following two reasons [42]: (i) The Hall–Petch effect and twinning-slip interaction are always enhanced by increased twin boundaries. Meanwhile, the twin boundaries are regarded as the barriers to certain slip systems. (ii) Compared to the parent grain under the same impact stress, the twinning form reoriented regions are easier to drive more slip or twin modes within twinned domains. Hence, the $\left\{10\bar{1}2\right\}$ tension twinning and non-basal slip simultaneously exist and compete with each other. When the temperature of the experiment is 250 °C, the direction of the c-axis is a completely departure from the normal direction of the wrought AZ31 alloy sheet after post impact, as shown in Figure 7c. Meanwhile, the CRSS of the prismatic slip and pyramidal <c + a> are directly dropped to be equivalent with $\left\{10\bar{1}2\right\}$ tension twinning, which is 40 MPa, as listed in Table 2. As the priority of CRSS and SF in $\left\{10\bar{1}2\right\}$ tension twinning almost disappeared, the non-basal slip and $\left\{10\bar{1}2\right\}$ tension twinning are activated almost at the same time. In addition, the grain refinement in gradually generated in the matrix of the wrought AZ31-TD alloy, as shown in Figure 8, which is caused by twin-induced dynamic recrystallization (TDRX) and continuous dynamic recrystallization (CDRX) [43].

In detail, the DRX can be induced by twins in the following three types: (i) the primary twin is mutually interacted, (ii) the secondary twin is initially nucleate and (iii) the twin is subdivided into nuclei, which is caused by the low angle grain boundaries inside coarse twin lamellae converting into high angle grain boundaries that depend on further increased strain. For CDRX, the rotational dynamic recrystallization (RDRX) of magnesium alloy is the most common mechanism. In the mechanism of RDRX, the local lattice rotation is completely activated, which is mainly caused by the transformation of the grain boundary from a low angle to a high angle grain boundary due to a dislocation accumulation. As a result, the stress–strain curve is a concave-down curve, as shown in Figure 2, which is caused by the effect of non-basal slip, $\left\{10\bar{1}2\right\}$ tension twinning and DRX simultaneously.

## 5. Conclusions

In the current paper, the effect of temperature (20–250 °C) on the dynamic compression behavior of the wrought AZ31-TD alloy under 1000 s^−1^ is investigated using a SHPB test and EBSD observation. The mechanical responses are reflected by stress–strain curves. The deformation mechanism evolution is analyzed and discussed based on CRSS analysis, SF calculation and microstructure observation. Finally, the major conclusions can be summarized as follows:

1. With the temperature elevated from 20 to 250 °C, the curve is gradually converted from a sigmoidal curve to an approximate concave-down curve. As a result of the thermal soften effect, the critical plastic strain is increased from 0.12 to 0.14. Additionally, the peak stress is decreased from 398.61 to 204.16 MPa.
2. During the dynamic deformation with an elevated temperature,$\;\left\{10\bar{1}2\right\}$ tension twins, $\left\{10\bar{1}1\right\}$ contraction twins and $\left\{10\bar{1}1\right\}-\left\{10\bar{1}2\right\}$ double twins are gradually decreased. Even the $\left\{10\bar{1}1\right\}$ contraction twins and $\left\{10\bar{1}1\right\}-\left\{10\bar{1}2\right\}$ double twins disappear at 150 °C. In addition, the refinement grains are initially nucleate at 150 °C and grow at 250 °C.
3. According to the combined CRSS and SF analysis, $\left\{10\bar{1}2\right\}$ tension twinning is the preferential and main deformation mechanism of the wrought AZ31-TD alloy due to the impact loading perpendicular to the c-axis at 25 °C. The deformation mechanism of non-basal slip and $\left\{10\bar{1}2\right\}$ tension twinning simultaneously exist and compete with each other at 150 °C. The DRX and non-basal slip are largely activated and enhanced at 250 °C, which is induced by $\left\{10\bar{1}2\right\}$ tension twinning.

## Figures and Tables

**Table 1:** Chemical composition of AZ31 magnesium alloy.

Al	Mn	Zn	Fe	Si	Be	Cu	Mg
3.19	0.334	0.81	0.005	0.02	0.01	0.005	Bal.

**Table 2:** The CRSS of different microstructure deformation mechanism [38,39,40,41].

Mechanism Mode	CRSS (MPa)		
	20 °C	150 °C	250 °C
basal slip	5	4	3
prismatic slip	90	60	40
pyramidal <c + a>	110	65	40
$\left\{10\bar{1}2\right\}$ tension twinning	30	32	40
$\left\{10\bar{1}1\right\}$ contraction twinning	200	180	≥130

## Data Availability

No new data were created or analyzed in this study. Data sharing is not applicable to this article.
